# Mastitis vaccines in dairy cows: Recent developments and recommendations of application

**DOI:** 10.14202/vetworld.2017.1057-1062

**Published:** 2017-09-12

**Authors:** Zuhair Bani Ismail

**Affiliations:** Department of Veterinary Clinical Sciences, Faculty of Veterinary Medicine, Jordan University of Science and Technology, Irbid 22100, Jordan

**Keywords:** dairy cows, immunization, mastitis, pathogens, udder

## Abstract

The objective of this review article was to summarize the most recent clinical field trials that have been published evaluating the use of different types of vaccines against mastitis pathogens in dairy cows. Mastitis is one of the most common and economically important diseases in dairy cows in the world. The disease is considered an important welfare issue facing the dairy industry in addition to the loss of production and premature removal or death of affected cows. Losses are also related to high cost of veterinary medicines and the cost of unsalable milk of treated cows. Mastitis can be caused by either contagious or environmental pathogens both of which are best prevented rather than treated. In addition to the application of best management practices in the parlor during milking, vaccination against common udder pathogens is widely practiced in many dairy farms to prevent or reduce the severity of clinical mastitis. In this review, the most recent clinical field studies that evaluated the use of different types of vaccines in dairy cows are summarized.

## Introduction

Mastitis is one of the most economically devastating diseases in dairy cattle worldwide [1,2]. It is also considered one of the most important diseases that affect the welfare of the animal on the farm. It has been estimated that more than $130 million ($200/cow/year) is lost every year because of mastitis in Australia. Economic losses due to mastitis are mostly due to losses milk production, premature culling or removal from the herd, unsalable or poor quality milk, cost of veterinary care and medicines [2]. Affected cows suffer from general ill health and poor reproductive performance [3]. The most commonly isolated pathogens from infected udders are *Staphylococcus aureus* (*S. aureus*), *Escherichia coli* (*E. coli*), *Streptococcus uberis* (*Strep. uberis*), and *Streptococcus dysgalactiae* (*Strep. dysgalactiae*) [4-6].

To improve the general health, welfare and productivity of dairy cows, many mastitis therapeutic and prevention strategies have been practiced over the years [7-10]. Vaccine development against common udder pathogens has been advancing in the past few decades. Both commercial vaccines and herd-specific autovaccines using killed whole bacterial cells are commonly used in dairy herds with less than satisfactory outcomes in most cases.

In this review, a summary of the most recent clinical field trials that have been published in scientific and refereed journals evaluating the use of different types of vaccines against mastitis pathogens in dairy cows is provided. Data engines such as Google Scholar, PubMed, and Research Gate were searched, and only published articles in refereed journals were selected. This review intended to provide the reader with most updated clinical data obtained from the field regarding the use of different commercially available and herd-specific vaccines against common udder pathogens in dairy cows.

## Types of Vaccines Used in Efficacy Trials

Review of the recent literature revealed 15 published scientific, clinical field and experimental challenge trials that evaluated different types of vaccines against mastitis pathogens in dairy cattle. In this review, vaccines were classified according to the field of use: (a) marketed vaccines, (b) autogenous (herd-specific) vaccines, and (c) experimental vaccines (Table-1). Regardless of the source of the pathogen, results of efficacy studies of these vaccines have been controversial or modest at best. The most commonly targeted udder pathogens were *S. aureus*, *S. agalactiae*, and *E. coli*. Vaccines against *S. aureus* and *S. agalactiae* contained either the whole organism (cellular lysates, inactive, and attenuated vaccines) or subunits (toxins, surface proteins, and polysaccharides) while for *E. coli*, the mutant core antigen J5 was used most commonly. Vaccines were also classified as mono or polyvalent according to the number of targeted pathogens it contained.

**Table-1:** Target pathogens, dosage, route of administration, and results/recommendations of different mastitis vaccine studies in dairy cows.

References	Target pathogens	Dose and route	Results/recommendations
Freick *et al*. [11]	*S. aureus* *E. coli*	2 ml, intramuscularly once	No effect on udder health parameters
Bradley *et al*. [12]	*S. aureus* *E. coli*	2 ml, intramuscularly at 45, 10 days before calving; 52 days after calving	No significant difference mastitis incidence Significant reduction in severity of clinical cases No significant difference culling rate Significantly more milk production
Landin *et al.* [13]	*S. aureus* *E. coli*	2 ml, intramuscularly at 45, 10 days before calving; 52 days after calving	No effect on udder health parameters
Schukken *et al*. [6]	*S. aureus* *E. coli*	2 ml, intramuscularly at 45, 10 days before calving; 52 days after calving	Moderate reduction in mastitis incidence Pronounced reduction in duration of mastitis
Middleton *et al*. [15]	*S. aureus*	5 ml, intramuscularly twice 14 days	No significant differences mastitis incidence No significant differences in cure rate
Wilson *et al*. [16]	*E. coli*	2 ml, subcutaneously at 60 and 28 days before calving	Significant increase in J5-specific IgG Significantly lower culling rate
Chang *et al*. [17]	*S. aureus*	5 ml intramuscularly 3 times at 2 weeks interval	Significantly lower SCC Less *S. aureus* isolated
Leitner *et al*. [18]	*S. aureus*	1 ml subcutaneously under the tail and 1 ml in the area of the supramammary lymph node Booster after 40-60 days	Significant increase in serum immunoglobulin Significantly lower milk SCC Significantly more milk production
Czernomysy-Furowicz *et al*. [19]	*S. aureus*	3 ml, supramammary lymph nodes area once Cefuroxime, intramammary every 12 h for 5 times	60% reduction in *S. aureus* in milk
Magaš *et al.* [20]	*S. aureus* *S. agalactiae*	5 ml, intramuscularly once	No significant changes in milk SCC Significant increase in milk immunoglobulin
Prenafeta *et al*. [21]	*S. aureus*	5 ml, intramuscularly twice before calving at 2 weeks intervals	Significantly serum and milk immunoglobulin Significant reduction in milk *S. aureus* count No significant effect mastitis on clinical signs
Slobodanka *et al.* [22]	*S. aureus*	5 ml, subcutaneously 60 and 30 days before calving	Significant decrease in mastitis rates
Lee *et al*. [23]	*S. aureus*	2 ml, intramuscularly 2 ml, supramammary lymph nodes area at 30 days before calving Booster twice at 2 week interval	Significant increase in serum immunoglobulin Significant increase in CD4+ and CD8+ lymphocytes Slightly increased neutrophil phagocytosis

SCC=Somatic cell counts, IgG=Immunoglobulin, *S. aureus=Staphylococcus aureus*, *S. agalactiae=Streptococcus agalactiae*, *E. coli=Escherichia coli*

## Study Designs and Monitoring Parameters

In the clinical field trials, vaccines were either tested using normal herds or in herds with high mastitis rates using adult cows or heifers. Monitoring parameters that were used most commonly in the assessment of vaccine efficacy studies are listed in Table-2. Parameters included bulk tank and quarter milk somatic cell counts (SCC), milk yield and milk constituents, milk bacterial culture, milk bacterial counts, incidence and severity of clinical mastitis cases, subclinical mastitis rates, cure rates of clinical and subclinical mastitis cases, cull rate or cow removal from the herd, cow survival, milk and serum specific immunoglobulin (IgG) concentrations, and neutrophil, and lymphocytes function tests.

**Table-2:** Monitoring parameters used most commonly to evaluate the effects of mastitis vaccines in dairy cows.

References	Monitoring parameters
Freick *et al*. [11]	Somatic cell count Milk bacterial culture Milk production Clinical mastitis cases incidence and severity
Bradley *et al*. [12]	Milk production Clinical mastitis cases incidence and severity
Landin *et al.* [13]	Somatic cell count Milk bacterial culture Milk production Clinical mastitis cases incidence and severity Cow survival
Czernomysy-Furowicz *et al.* [19]	Milk bacterial culture Clinical mastitis cases incidence and severity
Schukken *et al*. [6]	Clinical mastitis cases incidence and severity Cure rate
Magaš *et al*. [20]	Somatic cell count Milk IgG concentrations
Prenafeta *et al.* [21]	Somatic cell count Serum and milk IgG concentrations Clinical mastitis severity *S. aureus* count in milk
Middleton *et al.* [15]	Somatic cell count Milk bacterial culture Milk IgG concentrations Clinical mastitis cases incidence and severity
Wilson *et al*. [16]	J5- serum specific IgG concentrations Milk production Clinical mastitis cases incidence and severity Cull rate
Chang *et al.* [17]	Somatic cell count Milk bacterial culture Serum IgG concentrations
Slobodanka *et al.* [22]	Somatic cell count Milk IgG concentrations Clinical and subclinical mastitis incidence and severity
Lee *et al*. [23]	Serum IgG concentrations
Leitner *et al.* [18]	Somatic cell count Serum IgG concentrations Clinical mastitis cases severity

IgG=Immunoglobulin, *S. aureus=Staphylococcus aureus*

## Commercial Vaccines

The commercially available polyvalent vaccine (Startvac, Hipra, Spain) containing *E. coli* J5 and *S. aureus* strain SP 140 was evaluated in a dairy herd with elevated bulk milk SCC with *S*. *aureus* as the main udder pathogen causing the mastitis [11]. Pregnant heifers, as well as cows with various udder health status classifications, were vaccinated using the commercial vaccine and compared to herd mates that were vaccinated using containing a herd-specific vaccine containing *S. aureus* (Best Vac) or those unvaccinated control [11]. The prevalence of *S. aureus* mastitis was significantly lower in cows that were vaccinated using the commercial vaccine and those vaccinated using the herd-specific vaccine compared to the controls [11]. It was also noted that 305-day milk production was significantly less in vaccinated groups [11]. However, there were no significant differences in the incidence or duration of clinical mastitis cases in the herd [11]. It was concluded that the application of these two different vaccines was not an appropriate tool to improve various udder health and milk production parameters [11].

In another field clinical trial, the commercial vaccine (Startvac) was evaluated on commercial units under UK field conditions [12]. Cows from seven different dairy farms were randomly selected for the study and were administered the vaccine according to label recommendations [12]. In another group of cows, the vaccine was vaccinated every 90 days following the initial vaccination course, and the third group was left unvaccinated to act as controls [12]. In the first 120 days of lactation, there was no significant difference in the incidence or prevalence of clinical or subclinical mastitis between any of the three groups [12]. However, vaccination following the label recommendation resulted in a significant reduction in the severity of clinical mastitis [12]. It was also found that cows vaccinated using the label vaccine recommendations produced significantly more milk and more milk solids than unvaccinated cows [12]. A net return of investment of approximately 2.57:1 was suggested due to increased milk yield alone [12].

Startvac was also evaluated in another study [13]. In two large Swedish dairy herds, cows were vaccinated according to label recommendations [13]. Various udder health and milk production parameters and survival rates were analyzed during the first 120 days of lactation [13]. There were no significant differences between vaccinated and unvaccinated groups in any of parameters investigated [13]. This study concluded that vaccination using a commercial polyvalent vaccine was not beneficial in improving udder health, milk production or survival in two commercial dairy herds with mastitis problems due to *S. aureus* [13].

The commercial vaccine Startvac was again evaluated in reducing or preventing new clinical mastitis cases due to with *S. aureus* and coagulase-negative *Staphylococci* under field conditions [6]. Cows were monitored for 21 months, and the mastitis cure rate, the rate of new infections, the prevalence, and duration of infections were analyzed [6]. In this study, vaccination was found to result in a moderate reduction in the incidence of new mastitis cases due to *S. aureus* and coagulase-negative *Staphylococci*, and moreover, vaccination resulted in a pounced reduction in the duration of mastitis [6]. Furthermore, the effect of Startvac vaccine on the immune response of dairy cows and heifers after experimental intramammary challenge with a heterologous killed *S. aurous* strain was evaluated [14]. Vaccination resulted in lower SCC and polymorphonuclear cell counts and less drop in milk production compared to non-vaccinated animals. Vaccinated animals had significantly more serum specific *S. aureus* and J5 IgG concentrations [14].

In one study used to evaluate the efficacy of a commercially available *S. aureus* bacterin in protecting against *S. aureus* and coagulase-negative *Staphylococci* mastitis, milk SCC, and milk antibody concentrations were assessed in lactating cow model [15]. There were no significant differences in the rate of new mastitis cases or cure rates between vaccinated and unvaccinated cows [15]. They concluded that this commercial bacterin was in an effective option to protect cows against new mastitis infections due to *S. aureus* or coagulase-negative *Staphylococci* [15].

Gram-negative bacteria including *E. coli* and *Klebsiella*, *Enterobacter*, and *Citrobacter* spp. are important causes of environmental mastitis in dairy cattle [16]. A commercial vaccine containing *E. coli* rough mutant O111:B4 bacteria was used to evaluate the effect of vaccination on the incidence of clinical mastitis, milk production, and culling or death of cows [16]. J5-specific antibody responses were also evaluated in cows that developed severe or mild clinical mastitis. It was found that in vaccinated cows, the amounts of IgG1 and IgG2 were significantly higher than in control cows [16]. Cows that contracted mastitis lost significantly more milk than controls [16]. It was also noted that odds of cows being culled because of mastitis as well as other reasons were significantly lower for J5 vaccinated cows [16].

The protective effect of a recombinant *Staphylococcal* enterotoxin Type C mutant vaccine (MastaVac) against experimentally induced mastitis in cows was evaluated in one study [17]. In this study, subclinical mastitis was induced in cows by inoculation of 50 CFU of *S. aureus* strain 409 per quarter [17]. Cows were then vaccinated 3 times intramuscularly at 2 weeks intervals [17]. Vaccinated cows developed detectable antibodies approximately 4 weeks after immunization. The SCC in vaccinated cows were significantly lower than unvaccinated cows after challenge with *S. aureus* [17]. Furthermore, no *S. aureus* was isolated from milk of those cows that were vaccinated, whereas 75% of unvaccinated cows, the bacteria could be isolated from their milk [17]. This study indicated that this commercial vaccine could protect cows against *S. aureus* intramammary infection during lactation [17].

Another commercial vaccine mainly against *S. aureus* mastitis was evaluated in a large scale vaccination field trial [18]. Vaccinated heifers were followed up for 2 years following vaccination [18]. Antibodies were detected in all vaccinated cows as early as 4-5 weeks post-primary immunization and were sustained throughout the experimental period [18]. The SCC and milk production were significantly different between the vaccinated and unvaccinated groups [18]. These results suggested that this commercial vaccine elicited a non-specific health improvement of the udder in addition to specific protection against *S. aureus* [18].

## Herd-specific Autovaccines

A herd-specific autovaccine (Best Vac) was tested in both pregnant heifers and adult cows in dairy herds against *S. aureus* mastitis [11]. The results were also compared to cows that were vaccinated using a commercially available vaccine (Startvac) in the same herd [11]. The herd-specific vaccine resulted in a significant reduction in the prevalence of *S. aureus* mastitis in vaccinated cows similar to the results obtained by the commercial vaccine [11]. Milk production parameters were also significantly improved in vaccinated cows [11].

In another study, the efficacy of a herd-specific autovaccine alone or in combination with intramammary antibiotic therapy using cefuroxime in eliminating *S. aureus* infection in a herd with high subclinical mastitis rate was evaluated [19]. It was concluded that the combination of the antibiotic/autovaccine was an effective method to eliminate subclinical mastitis problems due to *S. aureus* in this herd [19]. It was claimed that clinical mastitis was not recorded in vaccinated cows for at least 2 years [19].

The efficacy of a polyvalent herd-specific autovaccine containing *S. aureus* strain SAU 7 and *S. agalactiae* strain SAG 3 was evaluated [20]. The IgG response in the milk of vaccinated cows was significantly higher than in the milk of unvaccinated cows [20].

A vaccine contained the slime-associated antigenic complex was evaluated in an experimental model of clinical mastitis using primiparous pregnant cows [21]. Following vaccinations, the cows were challenged by intramammary infusion of virulent strain of *S. aureus* [21]. Vaccination of cows in this study resulted in a significant increase in serum and milk antibody titers and a significant reduction in *S. aureus* count in milk [21]. However, researchers did not find any significant differences in the severity of clinical signs of mastitis in challenged cows [21].

In another study, a herd-specific autovaccine was prepared from *S. aureus* JR3 cells and SM capsule of the strain *S. aureus* 2286 [22]. The vaccine resulted in significant reduction in the rate of subclinical and clinical mastitis in the vaccinated cows [22].

A trivalent vaccine contained *S. aureus* capsular polysaccharide type 5 (T5), 8 (T8), and 336 (T336) was evaluated in a case–control study in heifers [23]. Vaccination was resulted in a significant increase in serum antibody titers, significant increase in percentages of CD4+ and CD8+ lymphocytes and a slightly increase in neutrophil phagocytosis functions [23]. The researchers in this study suggested that the use of this whole cell trivalent vaccine may be able to induce a protective antibody response against the three capsular polysaccharide antigens of *S. aureus* [23].

In another study where a vaccine composed of three field isolates of *S. aureus* that were obtained from naturally occurring mastitis cases was evaluated in normal cows in an experimentally induced mastitis model [14]. There was an estimated 70% protection from infection in vaccinated cows compared to <10% in unvaccinated cows [14]. Clinical signs of induced mastitis were mild in vaccinated experimentally infected cows [14].

## Conclusions

Mastitis remains one of the most economically devastating diseases in dairy cows. Vaccination is one tool that could be used to prevent mastitis. However, regardless of the type of vaccine used, it alone is not necessarily effective or economical especially in dairy herds with high mastitis rates. The combination of vaccination and the application of other infection control procedures, such as excellent milking hygiene procedures, treatment of clinical cases, segregation, and culling of known infected cows are important preventative measures that usually result in a significant reduction in the incidence and duration of intramammary infections.

## Competing Interests

The author declares that he has no competing interests.

## References

[1] Nielsen C, Østergaard S, Emanuelson U, Andersson H, Berglund B, Strandberg E (2010). Economic consequences of mastitis and withdrawal of milk with high somatic cell count in Swedish dairy herds. Animal.

[2] Hogeveen H, Huijps K, Lam T.J (2011). Economic aspects of mastitis: New developments. N. Z. Vet. J.

[3] Dobson H, Walker S.L, Morris M.J, Routly J.E, Smith R.F (2008). Why is it getting more difficult to successfully artificially inseminate dairy cows?. Animal.

[4] Unnerstad H.E, Lindberg A, Waller K.P, Ekman T, Artursson K, Nilsson-Ost M, Bengtsson B (2009). Microbial etiology of acute clinical mastitis and agent-specific risk factors. Vet. Microbiol.

[5] Persson Y, Nyman A.K, Grönlund-Andersson U (2011). Etiology and antimicrobial susceptibility of udder pathogens from cases of subclinical mastitis in dairy cows in Sweden. Acta Vet. Scand.

[6] Schukken Y.H, Bronzo V, Locatelli C, Pollera C, Rota N, Casula A, Testa F, Scaccabarozzi L, March R, Zalduendo D, Guix R, Moroni P (2014). Efficacy of vaccination on *Staphylococcus aureus* and coagulase-negative *Staphylococci* intramammary infection dynamics in 2 dairy herds. J. Dairy Sci.

[7] Barkema H.W, Schukken Y.H, Zadoks R.N (2006). The role of cow, pathogen, and treatment regimen in the therpeutic success of bovine *Staphylococcus aureus* mastitis. J. Dairy Sci.

[8] Pérez M.M, Prenafeta A, Valle J, Penadés J, Rota C, Solano C, Marco J, Grilló M.J, Lasa I, Irache J.M, Maira-Litran T, Jiménez-Barbero J, Costa L, Pier G.B, de Andrés D, Amorenaa B (2009). Protection from *Staphylococcus aureus* mastitis associated with poly-N-acetyl β-1,6 glucosamine specific antibody production using biofilm-embedded bacteria. Vaccine.

[9] Pereira U.P, Oliveira D.G.S, Mesquita L.R, Costa G.M, Pereira L.J (2011). Efficacy of *Staphylococcus aureus* vaccines for bovine mastitis: A systematic review. Vet. Microbiol.

[10] Keefe G (2012). Update on control of *Staphylococcus aureus* and *Streptococcus agalactiae* for management of mastitis. Vet. Clin. N. Am. Food Anim. Pract.

[11] Freick M, Frank Y, Steinert K, Hamedy A, Passarge O, Sobiraj A (2016). Mastitis vaccination using a commercial polyvalent vaccine or a herd-specific *Staphylococcus aureus* vaccine. Results of a controlled field trial on a dairy farm. Tierarztl. Prax.

[12] Bradley A.J, Breen J.E, Payne B, White V, Green M.J (2015). An investigation of the efficacy of a polyvalent mastitis vaccine using different vaccination regimens under field conditions in the United Kingdom. J. Dairy Sci.

[13] Landin H, Jansson M, Larsson M.M, Waller K.P (2015). Vaccination against *Staphylococcus aureus* mastitis in two Swedish dairy herds. Acta Vet. Scand.

[14] Piepers S, Prenafeta A, Verbeke J, de Visscher A, March R, de Vliegher S (2016). Immune response after an experimental intramammary challenge with killed *Staphylococcus aureus* in cows and heifers vaccinated and not vaccinated with Startvac, a polyvalent mastitis vaccine. J. Dairy Sci.

[15] Middleton J.R, Luby C.D, Adams S.D (2009). Efficacy of vaccination against *Staphylococcal* mastitis: A review and new data. Vet. Microbiol.

[16] Wilson D.J, Mallard B.A, Burton J.L, Schukken Y.H, Grohn Y.T (2009). Association of *Escherichia coli* J5-specific serum antibody responses with clinical mastitis outcome for J5 vaccinate and control dairy cattle. Clin. Vaccine Immunol.

[17] Chang B.S, Moon J.S, Kang H.M, Kim Y.I, Le H.K, Kim J.D, Lee B.S, Koo H.C, Park Y.H (2008). Protective effects of recombinant *Staphylococcal* enterotoxin Type C mutant vaccine against experimental bovine infection by a strain of *Staphylococcus aureus* isolated from subclinical mastitis in dairy cattle. Vaccine.

[18] Leitner G, Yadlin N, Lubashevsky E, Ezra E, Glickman A, Chaffer M, Winkler M, Saran A, Trainin Z (2003). Development of a *Staphylococcus aureus* vaccine against mastitis in dairy cows. II. Field trial. Vet. Immunol. Immunopathol.

[19] Czernomysy-Furowicz D, Fijalkowski K, Silecka A, Karakulska J, Nawrotek P, Drozd R, Ferlas M, Borkowski J, Jankowiak D (2014). Herd-specific autovaccine and antibiotic treatment in elimination of *Staphylococcus aureus* mastitis in dairy cattle. Turk. J. Vet. Anim. Sci.

[20] Magaš V, Slobodanka V, Pavlovic V, Velebit B, Mirilovic M, Maletic M, Duric M, Svetkana N (2013). Effiency evaluation of a bivalent vaccine in the prophylaxis of mastitis in cows. Acta Vet. (Beograd).

[21] Prenafeta A, March R, Foix A, Casals I, Costa L (2010). Study of the humoral immunological response after vaccination with a *Staphylococcus aureus* biofilm-embedded bacterin in dairy cows: Possible role of the exopolysaccharide specific antibody production in the protection from *Staphylococcus aureus* induced mastitis. Vet. Immunol. Immunopathol.

[22] Slobodanka V, Pavlović M, Pavlović V, Sonja O (2008). Immunoprophylaxis of *Staphylococcus aureus* mastitis in dairy cows. Acta. Vet. (Beograd).

[23] Lee J.W, O'Brien C.N, Guidry A.J, Paape M.J, Shafer-Weaver K.A, Zhao X (2005). Effect of a trivalent vaccine against *Staphylococcus aureus* mastitis lymphocyte subpopulations, antibody production, and neutrophil phagocytosis. Can. J. Vet. Res.

